# Physiological Modeling of Responses to Upper Versus Lower Lobe Lung Volume Reduction in Homogeneous Emphysema

**DOI:** 10.3389/fphys.2012.00387

**Published:** 2012-10-01

**Authors:** Arschang Valipour, Mordechai R. Kramer, Franz Stanzel, Axel Kempa, Sherwin Asadi, Oren Fruchter, Ralf Eberhardt, Felix J. Herth, Edward P. Ingenito

**Affiliations:** ^1^Department of Respiratory and Critical Care Medicine, Ludwig-Boltzmann-Institute for COPD, Otto Wagner HospitalVienna, Austria; ^2^Beilinson Hospital, Rabin Medical CenterPetah Tikva, Tel Aviv, Israel; ^3^Lungenklinik Hemer, Pneumologie – Thorakale EndoskopeHemer, Germany; ^4^Thoraxklinik am Universitätsklinikum HeidelbergHeidelberg, Germany; ^5^Brigham and Women’s HospitalBoston, MA, USA

**Keywords:** physiological modeling of emphysema, lung volume reduction

## Abstract

**Rationale:** In clinical trials, homogeneous emphysema patients have responded well to upper lobe volume reduction but not lower lobe volume reduction. **Materials/Methods:** To understand the physiological basis for this observation, a computer model was developed to simulate the effects of upper and lower lobe lung volume reduction on RV/TLC and lung recoil in homogeneous emphysema. **Results:** Patients with homogeneous emphysema received either upper or lower lobe volume reduction therapy based on findings of radionucleotide scintigraphy scanning. CT analysis of lobar volumes showed that patients undergoing upper (*n* = 18; −265 mL/site) and lower lobe treatment (LLT; *n* = 11; −217 mL/site) experienced similar reductions in lung volume. However, only upper lobe treatment (ULT) improved FEV_1_ (+11.1 ± 14.7 versus −4.4 ± 15.8%) and RV/TLC (−5.4 ± 8.1 versus −2.4 ± 8.6%). Model simulations provided an unexpected explanation for this response. Increases in transpulmonary pressure subsequent to volume reduction increased RV/TLC in upper lobe alveoli, while caudal shifts in airway closure decreased RV/TLC in lower lobe alveoli. ULT, which eliminates apical alveoli with high RV/TLC values, lowers the average RV/TLC of the lung. Conversely, LLT, which eliminates caudal alveoli with low RV/TLC values, has less effect. **Conclusion:** LLT in homogeneous emphysema is uniformly less effective than ULT.

## Introduction

Surgical lung volume reduction (LVRS) in patients with homogeneous emphysema has been shown to improve physiology and functional outcomes, although responses are generally smaller than in patients with heterogeneous upper lobe disease (Weder et al., 2009). Independent of the distribution of disease in any given patient, LVRS generally involves resection of tissue in the upper lobes due to limited surgical access to lower lobe sites (DeCamp et al., 2008). AeriSeal^®^ Emphysematous Lung Sealant (ELS) is a novel endoscopic lung volume reduction (ELVR) treatment shown to improve pulmonary function in patients with advanced emphysema (Herth et al., 2011a). In contrast to LVRS, ELVR therapies such as ELS allow physicians to access sites in the lower lobes endoscopically, facilitating lower lobe treatment (LLT).

While ELS improves lower lobe access, physiological benefit in homogeneous emphysema patients has been limited almost exclusively to those treated in the upper lobes. This study describes the development of a physiological model that explains why patients with advanced homogeneous emphysema who receive lower lobe ELVR are less likely to benefit than those treated in the upper lobes.

## Materials and Methods

### Study subjects

Results for the subset of patients with advanced homogeneous emphysema from two clinical trials of AeriSeal ELS treatment (#NCT01051258 and #NCT01181466) are included in this report (Herth et al., 2011a,b). Both trials were open-labeled and multi-center, and were performed at four teaching hospitals in Germany, one in France, one in Austria, and two in Israel. Studies were approved by the Ethics Committee’s at all participating centers. Written informed consent was obtained from all patients whose data is included in this manuscript. Patient inclusion/exclusion criteria are listed on www.clinicaltrials.gov.

Thirty patients with advanced homogeneous emphysema were enrolled in study NCT01051258. All participants were treated at two subsegmental sites unilaterally. Eighteen received upper lobe treatment (ULT), and 12 LLT. Three month follow-up data, including physiology and CT imaging, is available for 29 patients, 18 in the ULT group and 11 in the LLT group.

Ten patients with advanced homogeneous emphysema were included in study NCT011811466, which was initiated following completion and analysis of data from study NCT01051258. All patients in this second study received ULT at four subsegments, two in each upper lobe. Three month follow-up data including physiology and CT imaging is available for nine patients.

### Design

Screening evaluations in both studies included pulmonary function tests (spirometry, plethysmography, and single breath diffusing capacity), functional and quality of life assessments, chest CT scan performed at full inspiration, and quantitative scintigraphy perfusion scanning.

### Methods – image acquisition and analysis

CT images were generated using a standardized acquisition/reconstruction algorithm (spiral acquisition using a multi-detector CT scanner with 0.75–1 mm collimation, pitch of 1, and 0.5 mm overlap). Computer analysis of baseline and post treatment images was performed using commercially available software (VIDA Diagnostics Pulmonary Workstation Plus Software, Iowa City, IA, USA) to confirm homogeneous emphysema among study patients, and to measure lobar volume reduction in response to ELS treatment at 3 month follow-up. Disease heterogeneity was evaluated quantitatively, and expressed as a Heterogeneity Index (HI), defined as the ratio of % of voxels with a density <−910 HU in the upper lobes (right upper + left upper) to that in the lower lobes (right lower + left lower). Patients were designated as having homogeneous disease if their HI was between 0.85 and 1.15. ELS treatment sites were chosen based upon quantitative scintigraphy perfusion scanning, targeting regions with low perfusion.

Baseline and 3 month follow-up physiology and radiology results were used to assess disease distribution, lobar volume reduction, and corresponding physiological responses to treatment.

### Methods – computer modeling

A computer model was developed to simulate the effects of targeted volume reduction on gas trapping. The model can be used to examine physiological response patterns in either heterogeneous or homogeneous disease but was used solely for simulating homogeneous physiology in this analysis.

The lung was modeled as a collection of discrete alveoli arranged in layers from diaphragm to apex. The healthy lung was considered to have 300 million alveoli. Alveolar number (*n*) and size were independent simulation parameters. Each alveolus was modeled as having a constitutive pressure-volume relationship of the form *V = V*_max_−*Ae^−kPtp(z)^*, where *V* equals alveolar volume, *P*_tp_(*z*) transpulmonary pressure as a function of cranio-caudal location (*z*), *A* = *V*_max_−V_min_, *V*_max_ equals alveolar volume at “infinite” inflation pressure, *V*_min_ alveolar volume at 0 inflation pressure, and *k* the “shape factor” that determines the curvature of the exponential relationship between pressure and volume (Ingenito et al., 2003). *V*_max_, *V*_min_, *k*, and *P*_tm′_ are user-specified model parameters, and can vary from region to region to simulate heterogeneity in tissue damage. Since each alveolus is considered separately, *V*_max_, *V*_min_, *k*, and *P*_tm′_ could be considered larger for upper lobe alveoli than lower lobe alveoli when simulating upper lobe predominant emphysema. Conversely, the opposite would apply when simulating lower lobe predominant emphysema. However, for the simulations performed in this analysis of homogeneous emphysema, values of *V*_max_, *V*_min_, *k*, and *P*_tm′_ were considered as independent of location within the lung. *P*_tp_ varies as a function of distance (*z*) from the lung apex, and is equal to *P*_tp_(0)−ρ_lung_(*z*) × *g*, where *P*_tp_(0) equals *P*_tp_ at the apex, lung tissue density (ρ_lung_) is set equal to 25% of water density, and *g* is gravitational acceleration. Calculations were performed in Microsoft Excel^®^ to predict: (1) alveolar volumes at full inflation (Total Lung Capacity = TLC) and full deflation (Residual Volume = RV) as a function of cranio-caudal position within the lung; (2) regional alveolar RV/TLC ratios; and (3) overall RV, TLC, and RV/TLC values for the entire lung by integrating results across all alveoli.

Emphysema was modeled by reducing the total number of alveoli (*n*), increasing the size of alveoli (i.e., increasing *V*_max_ and *V*_min_), increasing alveolar gas trapping (i.e., increasing *V*_min_/*V*_max_), increasing *k* (to simulate iso-volume loss of recoil), and increasing *P*_tm′_ (to simulate premature airway closure and gas trapping due to loss of airway tethering). For the current simulations of homogeneous emphysema, *P*_tm′_ was considered invariant as a function of distance from the lung apex (i.e., *P*_tm′_ is constant throughout the lung), while *P*_tp(*z*)_ decreases monotonically from apex-to-base in the gravitational field. Therefore, near the lung apex *P*_tp_ exceeds *P*_tm′_ and airways remain opened. However, near the lung base, *P*_tm′_ can exceed *P*_tp_, and airways can close, trapping gas behind them. The location at which *P*_tp_ = *P*_tm′_ denotes the position (*z*) at which airway closure occurs. For alveoli located above this point, volume is determined by the equation, *V = V*_max_−*Ae^−kPtp(z)^*, and those alveoli nearer the lung apex are more inflated than alveoli nearer the diaphragm. Below the point at which *P*_tp_ = *P*_tm′_, alveolar volumes are considered invariant with position (*z*), and are set equal to the amount of trapped gas at airway closure.

The effects of ELS volume reduction therapy were simulated by: (1) reducing the number of alveoli at specific target sites (i.e., either upper or lower lung fields) to reflect elimination of alveolar units; (2) decreasing *P*_tm′_ to reflect the effects of parenchymal tethering on airway closing pressure; and (3) increasing transpulmonary pressure [*P*_tp_(*z*)] in proportion to the extent of volume reduction. Regional and global RV, TLC, and RV/TLC predicted by the model were then reassessed without changing model parameters of unaffected alveoli.

### Analysis, data presentation, and statistics

Spirometry and lung volume measurements of participants in studies NCT01051258, and NCT01181466 at baseline and following treatment are presented as mean ± standard deviation. The effect of ELS treatment is summarized as absolute and percentage change from baseline. Statistical significance of changes from baseline for continuous measures was assessed by paired *t* test. Comparisons across 3 or more subgroups were performed by one way analysis of variance. Assessment of statistical differences in categorical outcome measures between groups was assessed by Chi squared testing or Fisher’s exact test. Correlations were performed using the method of Pearson. Statistical significance was based on *P* values subject to correction for multiple comparisons using the method of Bonferroni.

## Results

### Baseline demographics, medical history, and pulmonary function

A total of 40 patients with homogeneous emphysema were enrolled in studies NCT01051258 (*n* = 30) and NCT01181466 (*n* = 10). Complete follow-up results are available for 38. Baseline characteristics, including demographics, smoking history, body mass index (BMI), GOLD classification, and medication use are summarized in Table 1. Results are presented for the entire group, for the 18 NCT01051258 ULT patients, the 11 NCT01051258 LLT patients, and the 9 NCT01181466 patients all of whom were treated in the upper lobes.

**Table 1:** **Summary of patient demographics and medical therapy**.

	All homogeneous patients	ULT NCT01051258	LLT NCT01051258	ULT NCT01181466	Group Difference *P* value
**Demographics**
Number of patients	38	18	11	9	–
Gender (males)	21	11	4	6	0.315*
GOLD stage III/IV	22/16	10/8	7/4	5/4	0.901*
Smoking hx (pk yrs)	43.7	47.9	38.8	42.7	0.674**
Age (yrs)	62 ± 8	64 ± 7	58 ± 9	63 ± 7	0.275**
BMI (kg/m^2^)	25.2 ± 3.9	25.3 ± 4.1	24.9 ± 3.7	25.1 ± 4.6	0.345**
**Medications**
SABA	31	15	9	7	0.940*
LABA	38	18	11	9	1.0*
SAAC	22	9	7	6	0.349*
LAAC	38	18	11	9	1.0*
ICS	29	13	9	7	0.835*
Theophylline	9	4	3	2	0.950*
Oral steroids	5	3	1	1	0.824*
Oxygen use (any)	17	8	5	4	0.998*

*Comparison of ULP NCT01051258, LLT NCT 01051258, and ULT 01181466 by 1-way Pearson Chi-Square for >2 groups.

**Comparison of ULP NCT01051258, LLT NCT 01051258, and ULT 01181466 by 1-way ANOVA.

Baseline characteristics were similar across all three subgroups with respect to age, gender distribution, BMI, smoking history, medication use, and disease severity. All patients were using inhaled beta-agonist (31 short acting, 38 long acting) and anticholinergic agent therapy (22 short acting, 38 long acting), 29 were using inhaled corticosteroids, 9 theophylline preparations, 5 oral corticosteroids, and 7 mucolytic agents. Seventeen patients were receiving domiciliary oxygen therapy.

Baseline pulmonary function for the entire group (*n* = 38), and for each subgroup is summarized in Table 2. Profiles are consistent with advanced emphysema, showing severe airflow obstruction and hyperinflation. Comparison across the groups (ANOVA) shows no significant differences in baseline pulmonary function.

**Table 2:** **Baseline patient characteristics**.

	Baseline physiology				
	All patients (*n* = 38)	ULT NCT01051258 (*n* = 18)	LLT NCT01051258 (*n* = 11)	ULT NCT01181466 (*n* = 9)	Group difference *P* value*
FEV_1_	0.93 ± 0.30 L (32.1 ± 8.3% pred; range: 20.5–54.2%)	0.93 ± 0.34 L (32.0 ± 9.7% pred; range: 20.5–54.2%)	0.94 ± 0.25 L (32.3 ± 5.4% pred; range: 24.1–39.6%)	0.95 ± 0.30 L (31.7 ± 5.4% pred; range: 20.1–39.0%)	0.985
FVC	2.53 ± 0.75 L (66.0 ± 14.5% pred; range: 39.0–90.5%)	2.55 ± 0.74 L (66.1 ± 15.4% pred; range: 39.0–90.5%)	2.50 ± 0.80 L (65.9 ± 13.4% pred; range: 51.3–86.6%)	2.89 ± 0.85 L (69.0 ± 14.6% pred; range: 42.6–94.3%)	0.456
RV	4.82 ± 1.13 L (221.2 ± 47.1% pred; range: 141–308%)	4.82 ± 1.06 L (215.0 ± 52.1% pred; range: 141–308%)	4.82 ± 1.30 L (232.9 ± 35.0% pred; range: 155–279%)	5.11 ± 0.95 L (217.5 ± 44.2% pred; range: 146–305%)	0.747
TLC	7.43 ± 1.25 L (127.2 ± 16.4% pred; range: 99–156%)	7.47 ± 0.87 L (125.6 ± 18.3% pred; range: 99–156%)	7.36 ± 1.82 L (130.1 ± 12.3% pred; range: 107–145%)	7.99 ± 1.31 L (123.8 ± 14.4% pred; range: 99–143%)	0.653

*Comparison between ULP NCT01051258, LLT NCT 01051258, and ULT 01181466 by 1-way ANOVA.

### Summary of overall physiological and functional outcomes

CT analysis confirmed the presence of homogeneous emphysema among study patients. The HI for the 38 patients in this analysis was (1.12 ± 0.26; Range 0.87–1.15). HI values for ULT patients in study NCT01051258 (1.10 ± 0.22; Range 0.92–1.15), LLT patients in study NCT01051258 (1.03 ± 0.34; Range 0.87–1.07), and patients in study NCT01181466 (1.16 ± 0.34; Range 1.03–1.15) were not significantly different (ANOVA; *p* = 0.445).

Quantitative CT analysis showed that LLT was associated with a −217 ± 125 mL/treatment lobar volume reduction, and a corresponding 35 ± 32 mL/treatment increase in upper lobe volume. The net volume reduction following LLT measured by CT analysis was −182 ± 121 mL/treatment. ULT was associated with a −265 ± 164 mL/treatment lobar volume reduction, and corresponding 65 ± 17 mL/treatment increase in lower lobe volume. The net volume reduction following ULT measured by CT analysis was −201 ± 166 mL/treatment (*p* = 0.321; ULT versus LLT).

The effects of ELS treatment on pulmonary function in patients with advanced homogeneous emphysema are summarized in Table 3. In study NCT01051258, unilateral treatment was associated with improvements in FEV_1_ (+6.3 ± 16.5%; *p* = 0.061), FVC (+7.5 ± 14.4%; *p* = 0.013), and RV/TLC ratio (−4.5 ± 8.2%; *p* = 0.011). Patients who underwent ULT (ΔFEV_1_ = +11.1 ± 14.7%, *p* = 0.005; ΔFVC = +9.8 ± 13.8%, *p* = 0.008; and ΔRV/TLC = −5.4 ± 8.1, *p* = 0.011) experienced significant improvements compared to baseline, while patients who underwent LLT did not, despite equivalent lobar volume reduction. Reductions in overall gas trapping, measured physiologically as change from baseline in RV/TLC, were greater for ULT (ΔRV/TLC = −5.4 ± 8.1%) than LLT (ΔRV/TLC = −2.4 ± 8.6%) patients. Fourteen of 18 (78%) patients receiving ULT experienced reductions in RV/TLC while only 4 of 11 (36%) LLT patients experienced reductions (*p* = 0.07, Fishers exact 2-tailed test).

**Table 3:** **Summary of response to ELS therapy at week 12**.

	All homogeneous patients (*n* = 38)	ULT NCT01051258 (*n* = 18)	LLT NCT01051258 (*n* = 11)	ULT NCT01181466 (*n* = 9)	Group difference *P* value*
ΔFEV_1_	8.7 ± 22.1%, *P* = 0.026, 32% responders**	11.1 ± 14.7%, *P* = 0.005, 39% responders**	−4.4 ± 15.8%, *P* = 0.45, 9% responders**	21.9 ± 36.3%, *P* = 0.159, 44% responders**	0.063
ΔFVC	8.1 ± 16.5%, *P* = 0.008, 32% responders**	9.8 ± 13.8%, *P* = 0.008, 33% responders**	2.4 ± 15.3%, *P* = 0.672, 22% responders**	10.5 ± 24.2%, *P* = 0.293, 33% responders**	0.537
ΔRV/TLC	−5.3 ± 8.2%, *P* = 0.0008	−5.4 ± 8.1%, *P* = 0.012	−2.4 ± 8.6%, *P* = 0.448	−9.1 ± 7.4%, *P* = 0.030	0.331

*Comparison between ULP NCT01051258, LLT NCT 01051258, and ULT 01181466 by one-way.

**Responder definitions: ΔFEV_1_ and ΔFVC ≥12% increase from baseline.

Change from baseline in RV/TLC at 3 month follow-up correlated significantly with the magnitude of lobar volume reduction assessed by CT imaging in ULT patients (*r* = 0.54; *p* = 0.02), but not in LLT patients (*r* = 0.09, *p* > 0.1), indicating that ULT volume reduction was effective in reducing gas trapping, while LLT volume reduction was not.

Based upon these findings, all homogeneous emphysema patients subsequently enrolled in the second study (NCT01181466) received ULT. Results from this second study confirm the effectiveness of upper lobe ELS treatment in homogeneous emphysema. Bilateral 4-site therapy at 3 month follow-up was associated with improvements in spirometry and overall gas trapping (Table 3).

### Model simulations of upper versus lower lobe volume reduction

**Figure 1:**
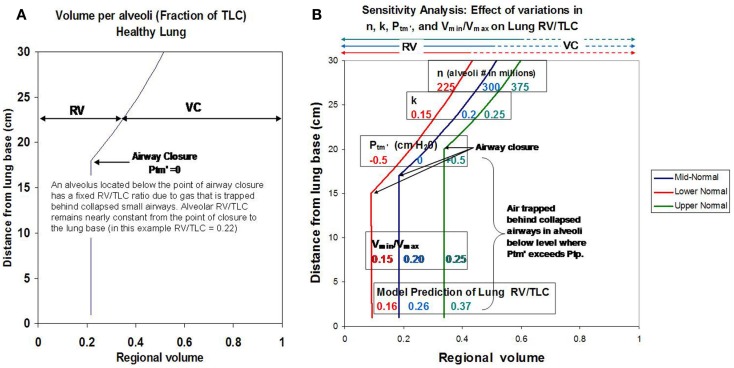
**(A)** Shows model simulations depicting RV/TLC ratios for individual alveoli in the healthy lung as a function of distance from the lung base. **(B)** Shows how changes in key model parameters (*n*, *k*, *V*_min_/*V*_max_, and *P*_tm′_) affect regional RV/TLC values when varied ±25% as a group from reported mean normal values.

Initial simulations were performed to validate the model by matching predicted results to RV/TLC values reported for healthy subjects (Anthonisen and Milic-Emili, 1966; Bryan et al., 1966; Behrakis et al., 1983). Using representative normal values for *n* (300 × 10^6^ alveoli), *V*_max_ (20 microliters/alveolus), *V*_min_ (4 microliters/alveolus), *k* (0.2), and *P*_tm′_ (0 cm H_2_O), model simulations to predict RV/TLC values for alveoli as a function of vertical distance from the lung apex were performed. Values were consistent with data reported by Milic–Emili in healthy young males (Figure 1A; Milic-Emili et al., 1966, 2007). A sensitivity analysis to evaluate the effects of systematic changes in model parameters was also performed. Varying parameters over a range considered representative of “normal” (i.e., ±25% of values for healthy lung) resulted in predicted RV/TLC values within the physiological range (Figure 1B).

**Figure 2:**
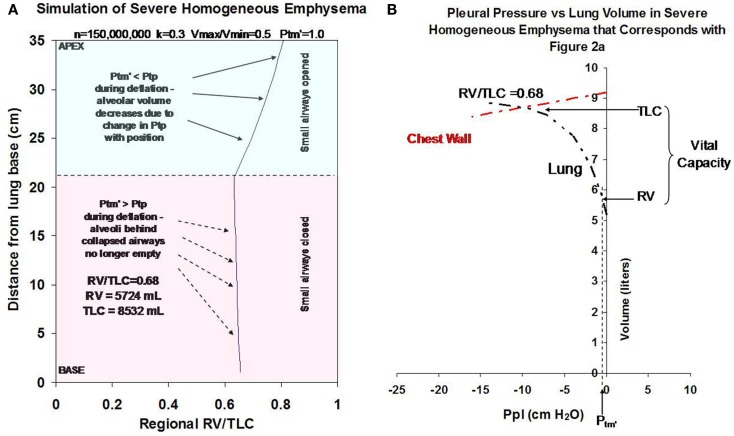
**(A)** Illustrates how changes in *V*_min_/*V*_max_ (from 0.25 to 0.5), *k* (from 0.2 to 0.3), *P*_tm′_ (from 0 to 1 cm H_2_O) and *n* (from 300,000,000 to 150,000,000 alveoli) to simulate severe homogeneous emphysema affect regional alveolar RV/TLC values as a function of distance from the lung base. **(B)** Illustrates how these changes affect gas trapping and recoil pressure for the overall lung. All simulations were performed assuming a 50% reduction in alveolar number from baseline (i.e., decrease from 300,000,000 to 150,000,000) and assume normal chest wall function (illustrated by red dashed line).

Simulations depicting advanced homogeneous emphysema are shown in Figures 2A,B. To simulate emphysema, the number of alveoli was set equal to 50% of normal (i.e., *n* = 150,000,000). The remaining model parameters (*V*_min_/*V*_max_, *k*, and *P*_tm′_) were also varied to simulate the effect of worsening emphysema. Moderate to severe tissue destruction was represented by an increase in *V*_min_/*V*_max_ from 0.25 to 0.50. Loss in tissue elasticity was represented by an increase in *k* from 0.2 to 0.3. An increase in airway closing pressure due to loss of airway tethering was represented by an increase in *P*_tm′_ from 0 to 1 cm H_2_O. Assuming a normal active chest wall compliance curve (red dashed line), the model predicts that these changes would cause marked hyperinflation, gas trapping, and a decrease in lung elastic recoil pressure as shown in Figure 2B. Such changes would be expected to cause marked airflow limitation (Ingenito et al., 2001, 2003).

**Figure 3:**
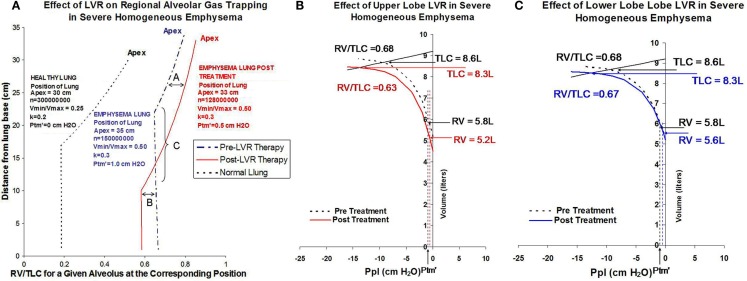
**(A)** Shows simulations depicting regional RV/TLC values for normal lung (black dashed line), severe homogeneous emphysema (blue dashed line), and following lung volume reduction therapy in homogeneous emphysema (red solid line) on regional RV/TLC values. Lung volume reduction therapy in this example is modeled as decreasing the overall size of the lung from 35 cm apex-to-base to 33 cm apex-to-base, decreasing the total number of alveoli from 150,000,000 to 128,000,000, and shifting airway closing pressure from 1 to 0.5 cm H_2_O. The increase in *P*_tp_ resulting from volume reduction therapy distends alveoli in the upper lung fields, increasing regional RV/TLC ratios, labeled in this figure as effect “A.” The decrease in *P*_tm′_ resulting from lung volume reduction therapy causes a caudal shift in the position of at which airway closure occurs, labeled in this figure as effect as “C.” Because lower lobe airways remain opened to a greater extent following lung volume reduction, they are able to deflate to a greater extent. This is labeled in this figure as effect “B.” **(B)** Simulates the effects of a 25% decrease in the number of alveoli in the upper lobes on overall gas trapping (RV/TLC) and recoil pressure. **(C)** Shows a corresponding simulation for the equivalent lower lobe volume reduction (elimination of the same number of alveoli). In this example, upper lobe volume reduction is associated with a 6% decrease in overall gas trapping, while lower lobe volume reduction is associated with a 1% decrease in overall gas trapping.

Using this model simulation of advanced homogeneous emphysema as a starting point, additional simulations were then performed to evaluate the physiological impact of upper lobe volume reduction and lower lobe volume reduction (Figure 3). The combined effects of an increase in recoil pressure at total lung inflation from 12.5 to 14 cm H_2_O, a shift in *P*_tm′_ from 1.0 to 0.5 cm H_2_O, and a decrease in cranio-caudal distance from apex to diaphragmatic dome from 35 to 33 cm while keeping *k* constant at 0.3 is predicted to have the following physiological impact:

1. increase RV/TLC for all alveoli remaining in the upper lung fields due to the distending effects of increasing *P*_tp_ from 12.5 to 14 cm H_2_O (labeled “A”);
2. decreasing RV/TLC values of alveoli in the lower lung fields due to the combined effects of gravity and a shift in *P*_tm′_ to a lower value (labeled “B”);
3. causing a caudal shift in the location at which airway closure occurs (labeled “C”).

The solid red line in Figure 3A shows how changes in transpulmonary pressure and changes in the position of *P*_tm′_ in the gravitation field resulting from volume reduction are predicted to affect gas tapping (i.e., RV/TLC) of any single alveolus as a function of its anatomic location. It does not, however, reflect how treatment would affect overall gas trapping (RV/TLC) or lung recoil, or how ULT versus LLT would differentially affect lung function. This requires combining the impact of changes in transpulmonary pressure and *P*_tm′_ with the effect of changing alveolar numbers regionally as a result of volume reduction. The effect of volume reduction treatment, in which either 25% of upper lobe alveoli or the equivalent number (20%) of lower lobe alveoli are removed, is shown in Figure 3B (ULT) and Figure 3C (LLT). In this example ULT is associated with a decrease in overall RV/TLC from 0.68 to 0.63 (7.3% reduction) while LLT is associated with minimal change (1.4%) despite a reduction in absolute RV from 5.8 to 5.6 L.

## Discussion

Conceptually, lung volume reduction therapy (LVRT) is a straightforward procedure that involves reducing the quantity of hyperinflated lung by eliminating damaged tissue. A simple but elegant physiological model to explain the effects of LVRT on airflow limitation in advanced emphysema was proposed over a decade ago by Fessler and Permutt (1998). This model has since been validated by clinical observations and physiological measurements (Ingenito et al., 2001; Fessler et al., 2002). Despite its near universal acceptance, the Fessler–Permutt model has at least one important limitation: by considering volume reduction therapy throughout the lung as equivalent, it fails to explain why patients who undergo volume reduction therapy in the upper and lower lobes respond differently.

The present study presents a novel perspective about the relative effects of upper versus lower lobe lung volume reduction on gas trapping in emphysema. While consistent with the Fessler–Permutt model, this analysis shows that gravitational effects can significantly affect physiological responses to volume reduction by attenuating the impact of LLT. Empirical observations from clinical trial NCT01051258 involving endoscopic ELS treatment in patients with homogeneous emphysema show that only patients treated in the upper lobes consistently experienced physiological benefit. This observation was then prospectively validated in a second study (NCT01181466) in which all patients received ULT. Although the number of patients in both studies was small, the results were highly consistent.

Differences in physiology between ULT and LLT patients following ELS were not due to treatment failure in the LLT cohort. Lobar volume reduction assessed by quantitative CT analysis was similar in the two groups. The impact of ELS on gas trapping assessed as change in RV/TLC measured by plethysmography, and as change in FEV_1_ measured by spirometry, was substantially different following ULT and LLT, however. Computer modeling indicates this is due to two factors not previously recognized as important when considering volume reduction therapy in this population: (1) lung volume reduction, independent of where it is performed, increases the RV/TLC ratio of alveoli in the upper lobes, but decreases RV/TLC ratio of alveoli in the lower lobes as shown in Figure 3A; and (2) ULT removes alveoli with the highest RV/TLC values, while LLT removes alveoli with lower RV/TLC values. As a result, ULT decreases both the number and mean RV/TLC value of alveoli remaining within the lung. Conversely, LLT decreases the number of remaining alveoli, but increases the mean RV/TLC value of remaining alveoli.

**Figure 4:**
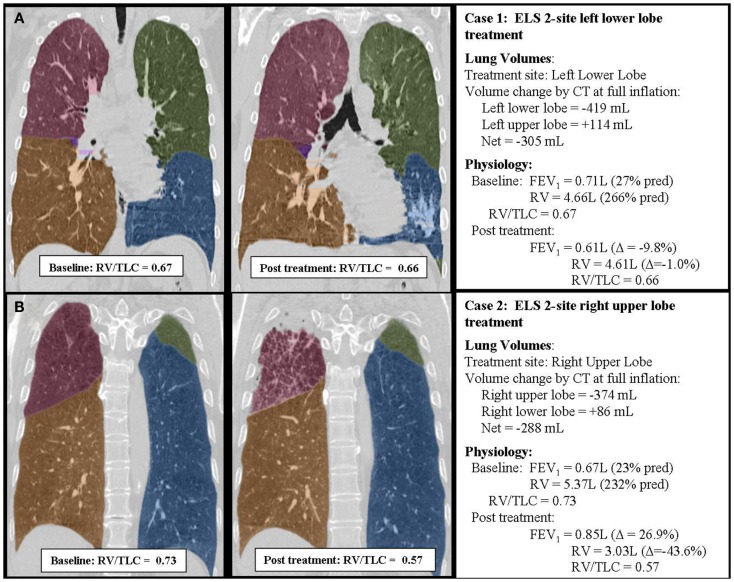
**Case summaries of two patients treated with ELS illustrating the differential effects of upper (B) and lower (A) lobe lung volume reduction**. The two cases were closely matched with respect to baseline patient characteristics, baseline physiology, and extent of lobar volume reduction measured by CT imaging. However, responses to treatment were quite different. Upper lobe treatment **(B)** was associated with a substantial improvement in lung function. Lower lobe treatment **(A)** had minimal beneficial effects on lung function. In each example, the individual lobes have been shaded to better define lobar anatomy: the right upper lobe is shaded red, the right middle lobe purple, the right lower lobe yellow, the left upper lobe green, and the left lower lobe blue. Note that physiology was measured in the upright position while CT imaging was performed supine. The supine position is expected to alter gravitational effects on CT measurements of lobar volume by underestimating upper lobe volumes and overestimating lower lobe volumes by up to approximately 10%.

The differential effect of ULT versus LLT will vary depending upon the baseline severity of gas trapping, the extent of the volume reduction, and the effects of treatment on *P*_tm′_ and *P*_tp_. Modeling indicates, however, that ULT will always be more effective than LLT in reducing RV/TLC in patients with homogenous emphysema. Figure 4 summarizes two clinical cases from study NCT01051258 that illustrate this point. These patients had similar baseline demographics, physiologic characteristics, and disease distribution patterns by CT imaging. One was treated in the left lower lobe and one in the right upper lobe based on baseline scintigraphy perfusion scanning showing hypoperfusion in these regions. Despite similar lobar volume reductions measured by quantitative CT imaging, overall physiological responses were markedly different, consistent with model predictions.

While these same physiological principles apply when considering LLT in patients with heterogeneous emphysema, the effects of treatment can be quite different (McKenna et al., 1997; Gelb et al., 1999; Chandra et al., 2010). The present analysis indicates that an important step in achieving therapeutic lung volume reduction is elimination of alveoli with “high RV/TLC” ratios, such that the mean RV/TLC of alveoli that remain in the lung post treatment is reduced. In heterogeneous emphysema, only the most damaged regions of lung identified by CT imaging are targeted for treatment, whether they reside in the upper or lower lobes. As a consequence, the mean RV/TLC ratio of alveoli remaining post treatment should always be reduced.

In summary, results from two small clinical trials show that lower lobe volume reduction using ELS is less effective than upper lobe volume reduction in patients with advanced homogeneous emphysema. Physiological modeling indicates that this is due primarily to differential effects of the procedure on upper versus lower lobe alveoli. Shifts in *P*_tp_ and *P*_tm′_ resulting from volume reduction therapy increase RV/TLC of upper lobe alveoli, while lowering RV/TLC of lower lobe alveoli. Removal of upper lobe alveoli reduces the mean RV/TLC of the alveoli remaining in the lung following therapy, while removal of lower lobe alveoli has the opposite effect. As a consequence, the beneficial effects of tissue resection following LLT are offset by an increase in gas trapped within the alveoli that remain. These findings suggest that LVRT for homogeneous emphysema should be limited to patients with upper lobe target sites.

## Author Contributions

Concept/study design – Arschang Valipour, Edward P. Ingenito, Mordechai R. Kramer; Clinical contributions – Arschang Valipour, Mordechai R. Kramer, Franz Stanzel, Axel Kempa, Sherwin Asadi, Oren Fructer, Ralf Eberhardt, Felix J. Herth; Manuscript preparation – Arschang Valipour, Edward P. Ingenito; Manuscript review – Arschang Valipour, Mordechai R. Kramer, Felix J. Herth, Ralf Eberhardt.

## Conflict of Interest Statement

Dr. Arschang Valipour – No conflicts of interest related to the data in this manuscript. Prof. Mordechai R. Kramer – No conflicts of interest related to the data in this manuscript. Dr. Franz Stanzel – No conflicts of interest related to the data in this manuscript. Dr. Axel Kempa – No conflicts of interest related to the data in this manuscript. Dr. Sherwin Asadi – No conflicts of interest related to the data in this manuscript. Dr. Oren Fruchter – No conflicts of interest related to the data in this manuscript. Dr. Ralf Eberhardt – No conflicts of interest related to the data in this manuscript. Prof. Felix J. Herth – No conflicts of interest related to the data in this manuscript. Dr. Edward Ingenito – A perceived conflict of interest exists for Dr. Ingenito with respect to the data in this manuscript. Dr. Ingenito is a senior medical advisor for Aeris Therapeutics, the company that funded the clinical trials that generated the data included in this study. Dr. Ingenito receives salary support from Aeris Therapeutics and owns stock in the company.
